# An update on the status of breast cancer screening in Brazil after the covid-19 pandemic

**DOI:** 10.11606/s1518-8787.2022056004545

**Published:** 2022-10-05

**Authors:** Jordana de Faria Bessa, Guilherme Novita, Ruffo Freitas

**Affiliations:** I Instituto D’Or de Ensino e Pesquisa São Paulo SP Brasil Instituto D’Or de Ensino e Pesquisa. Doutorado em Ciências da Saúde. São Paulo, SP, Brasil; II Sociedade Brasileira de Mastologia São Paulo SP Brasil Sociedade Brasileira de Mastologia. Regional São Paulo. São Paulo, SP, Brasil; III Universidade Federal de Goiás Departamento de Ginecologia e Obstetrícia Goiania GO Brasil Universidade Federal de Goiás. Departamento de Ginecologia e Obstetrícia. Goiania, GO, Brasil

**Keywords:** Breast Neoplasms, prevention & control, Mammography, statistics & numerical data, Women’s Health, trends, COVID-19

## Abstract

We have previously reported the impact of covid-19 pandemic on breast cancer screening, in Brazil: among women aged 50–69 years, mammography attendance decreased by 42% in public healthcare (SUS), comparing 2019 and 2020. In this short communication, we wish to present: a) an update of the number of mammograms performed, in 2021; b) an exploratory analysis of the characteristics of the screened population between 2019 and 2021. A total of 1.675.307 mammograms were performed in 2021, nearly 15% lower than pre-pandemic levels. Almost a third, 29.5% of them, had intervals greater than three years. In accordance with our previous study, the number of patients with palpable lumps on physical exam increased. The consequences of postponing breast cancer screening during the pandemic are still uncertain. Unfortunately, as of December 2021, screening attendance has not resumed. On the contrary, our results show an increase in the fraction of women with mammography delayed beyond three years.

We have previously reported the impact of the covid-19 pandemic on breast screening in Brazil^1^ . Among the target population (women aged 50–69 years), mammography attendance decreased by 42% in public healthcare (SUS), comparing 2019 and 2020.

Since the covid-19 pandemic outbreak, reports on its impact on cancer care have skyrocketed. For instance, as of January 2022, PubMed has 563 articles with keywords “Covid” and “breast cancer”. Most studies found a negative association between lockdown restrictions and breast cancer care, including difficult access to healthcare^2^ , increased prescription of preoperative endocrine therapy^3^ , and even delayed surgery beyond 12 weeks^4^ .

In this short communication, we wish to present: a) an update of the number of mammograms performed in the target screening population, in 2021; b) an exploratory analysis of the characteristics of the screened population between 2019 and 2021.

Just as for the first publication, we are interested in women aged 50–69 years and screened in public healthcare, according to data obtained from *Sistema de Informação do Câncer* (Siscan), a segment of Datasus, an open access database.

The results are presented in the Figure . A total of 1,675,307 mammograms were performed in 2021. This represents some recovery from 2020 (n = 1,190,577) but not to the extent seen in 2019 (n = 1,964,013). Therefore, 2021 attendance was still nearly 15% lower than pre-pandemic levels.

**Figure:**
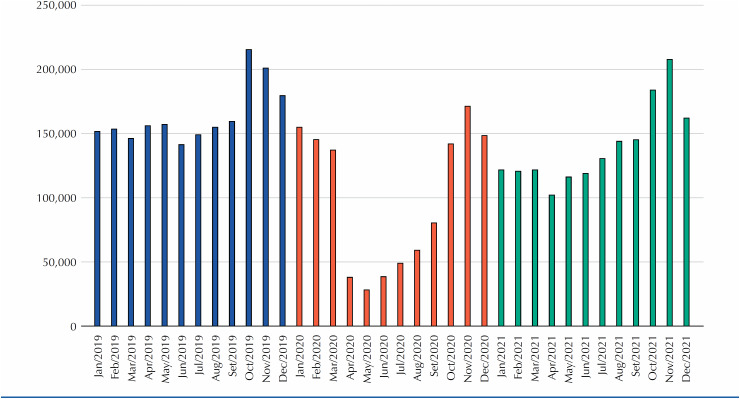
Mammograms from public healthcare (SUS) in women aged 50–69 years, 2019–2021, Brazil.

The partial recovery is probably a result of large-scale coronavirus vaccination campaigns and the subsequent contraction of the infection and mortality rates. Yet, it was not enough to increase the attendance to pre-pandemic levels. A reasonable sector of the population (especially those at higher risk for severe covid) possibly still has concerns about contracting covid. Also, screening units are adapted to reduce contamination, which means, among other measures, fewer patients scheduled by hour.

Table shows results stratified by year and subgroup. The proportion of patients with delayed exams sharply increased in 2021: almost a third of them had their last mammogram more than three years before. According to our previous paper, the number of patients with palpable lumps on physical exam increased. Finally, increased screening for elevated-risk patients was also a notable trend. Prioritizing such individuals (those with documented pathology of high-risk lesions, extremely dense breasts, personal history of thoracic radiation before 30 years old, strong familiar history for breast cancer and/or with deleterious pathogenic mutations) is a strategy to lessen the pandemic impact on cancer diagnosis, as most mammographic abnormal results are found in elevated-risk groups^5^ .

**Table 1:** Mammograms (SUS), in women aging 50–69 years, 2019–2021, Brazil.

Year		2019	2020	2021
Exams, total		1,964,013	1,190,577	1,675,307
Last exam (%)				
	Same year	3.59	3.26	3.04
	1 year	33.03	33.43	22.10
	2 years	25.31	25.36	29.28
	≥ 3 years	20.21	21.86	29.48
	Unknown	17.85	16.09	16.10
Screening population (%)				
	Average risk	93.56	92.14	92.41
	Elevated-risk (family history)	3.40	3.74	4.17
	Elevated-risk (personal history)	1.51	2.28	1.94
	Unknown	1.53	1.85	1.47
Palpable lump (%)				
	Yes	7.10	8.10	7.51
	No	92.90	91.90	92.49

Once again, we highlight the positive impact of the “Pink October” campaign on breast cancer awareness that resulted in a spike on the number of mammograms performed at the end of 2021.

The consequences of postponing breast cancer screening during the pandemic are still uncertain. As long as these patients return to regular screening, the impact might be minimal, since screening every year or every two years yield similar efficacy in average-risk women aged 50–69 years^6^ . But, unfortunately, at least as of December 2021, we have not seen screening attendance resuming. On the contrary, our results show an increase in the fraction of women with mammography delayed beyond three years.

The covid-19 pandemic might represent a wonderful opportunity to review and adapt breast cancer screening in countries with limited resources, like Brazil. Many authors claim that the greatest priority for Brazil is a one-stop clinic, which patients with palpable lumps or abnormal mammogram results could easily reach for a fast diagnosis, in contrast to the currently fragmented slow-paced care^7^ . We must prepare to provide excellent care for symptomatic patients if we want to also care for asymptomatic patients.
